# The genome sequence of the European chub, *Squalius cephalus *(Linnaeus, 1758)

**DOI:** 10.12688/wellcomeopenres.22579.1

**Published:** 2024-07-10

**Authors:** Richard Pitman, Bernd Hänfling, Darwin Tree of Life Barcoding collective, Wellcome Sanger Institute Tree of Life Management, Samples and Laboratory team, Wellcome Sanger Institute Scientific Operations: Sequencing Operations, Wellcome Sanger Institute Tree of Life Core Informatics team, Tree of Life Core Informatics collective, Darwin Tree of Life Consortium

**Affiliations:** 1 Environment Agency, Bristol, England, UK; 2 University of the Highlands and Islands, Inverness, Scotland, UK

**Keywords:** Squalius cephalus, European chub, genome sequence, chromosomal, Cypriniformes

## Abstract

We present a genome assembly from an individual *Squalius cephalus* (the European chub; Chordata; Actinopteri; Cypriniformes; Cyprinidae). The genome sequence is 1,101.9 megabases in span. Most of the assembly is scaffolded into 25 chromosomal pseudomolecules. The mitochondrial genome has also been assembled and is 16.61 kilobases in length.

## Species taxonomy

Eukaryota; Metazoa; Eumetazoa; Bilateria; Deuterostomia; Chordata; Craniata; Vertebrata; Gnathostomata; Teleostomi; Euteleostomi; Actinopterygii; Actinopteri; Neopterygii; Teleostei; Osteoglossocephalai; Clupeocephala; Otomorpha; Ostariophysi; Otophysi; Cypriniphysae; Cypriniformes; Cyprinoidei; Leuciscidae; Leuciscinae; *Squaliu*s; *Squalius cephalus* (Linnaeus, 1758) (NCBI:txid8284).

## Background

The European chub, *Squalius cephalus* (L.), is a widespread freshwater fish species found throughout Europe, excluding the Iberian Peninsula, Ireland, Northern Scandinavia, and Italy ( Freyhof, 2014). *S. cephalus* is a medium-sized omnivorous species that predominantly inhabits small and medium-sized rivers, with a preference for the barbel zone, but it can also be found in lentic habitats. The species' conservation status was last assessed by the IUCN in 2014 and is classified as "Least Concern" due to its broad geographic distribution and lack of major threats ( Freyhof, 2014). However, genetic analyses have revealed considerable intraspecific variation, highlighting the presence of distinct genetic lineages that correspond to geographical regions ( Durand *et al.*, 1999; Hänfling & Brandl, 1998; Seifertová *et al.*, 2012)

The genome of the European chub, *Squalius cephalus*, was sequenced as part of the Darwin Tree of Life Project, a collaborative effort to sequence all named eukaryotic species in the Atlantic Archipelago of Britain and Ireland. Here we present a chromosomally complete genome sequence for *Squalius cephalus*, based on one specimen from Calverton, Nottingham, UK.

## Genome sequence report

**Figure 1.**
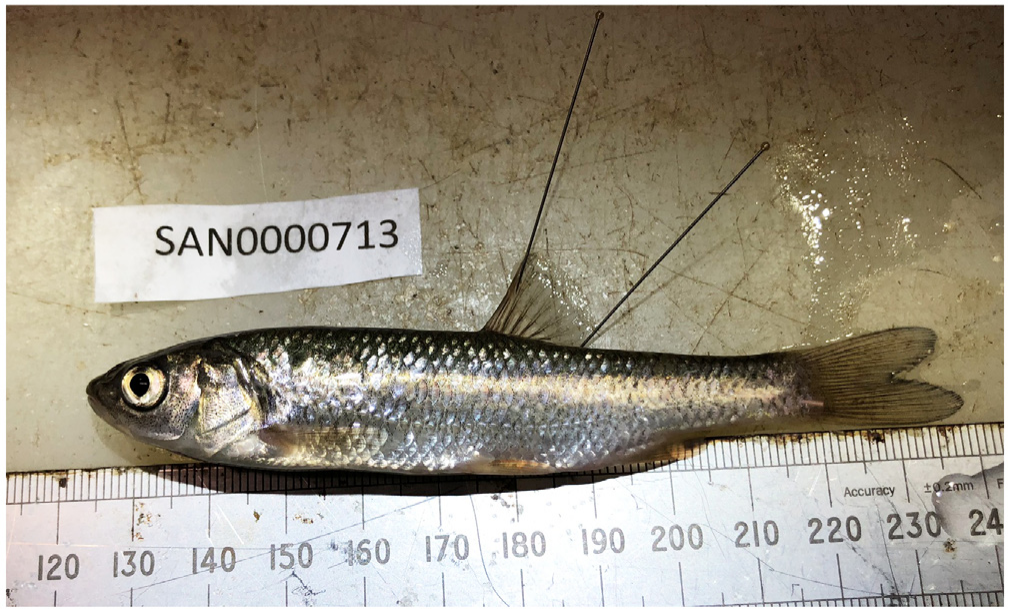
Photograph of the *Squalius cephalus* (fSquCep2) specimen used for genome sequencing.

The genome was sequenced from a *Squalius cephalus* ( Figure 1) collected from Calverton Fish Farm, Nottingham, UK (53.03, –1.05). A total of 46-fold coverage in Pacific Biosciences single-molecule HiFi long reads was generated. Primary assembly contigs were scaffolded with chromosome conformation Hi-C data. Manual assembly curation corrected 105 missing joins or mis-joins and removed 36 haplotypic duplications, reducing the assembly length by 2.30% and the scaffold number by 26.39%, and increasing the scaffold N50 by 9.50%.

**Figure 2.**
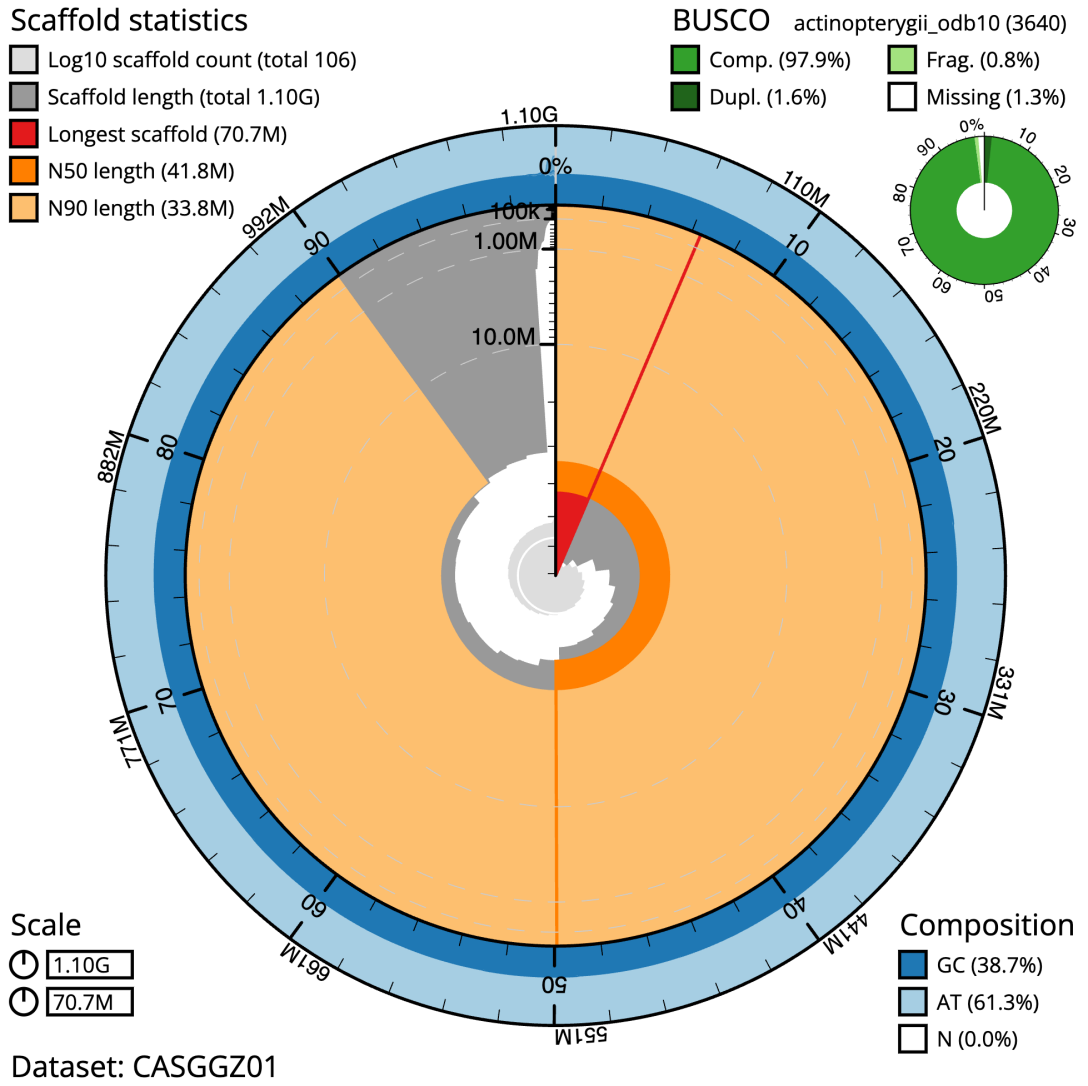
Genome assembly of *Squalius cephalus*, fSquCep2.1: metrics. The BlobToolKit Snailplot shows N50 metrics and BUSCO gene completeness. The main plot is divided into 1,000 size-ordered bins around the circumference with each bin representing 0.1% of the 1,101,930,522 bp assembly. The distribution of scaffold lengths is shown in dark grey with the plot radius scaled to the longest scaffold present in the assembly (70,742,303 bp, shown in red). Orange and pale-orange arcs show the N50 and N90 scaffold lengths (41,795,634 and 33,754,728 bp), respectively. The pale grey spiral shows the cumulative scaffold count on a log scale with white scale lines showing successive orders of magnitude. The blue and pale-blue area around the outside of the plot shows the distribution of GC, AT and N percentages in the same bins as the inner plot. A summary of complete, fragmented, duplicated and missing BUSCO genes in the actinopterygii_odb10 set is shown in the top right. An interactive version of this figure is available at https://blobtoolkit.genomehubs.org/view/CASGGZ01/dataset/CASGGZ01/snail.

**Figure 3.**
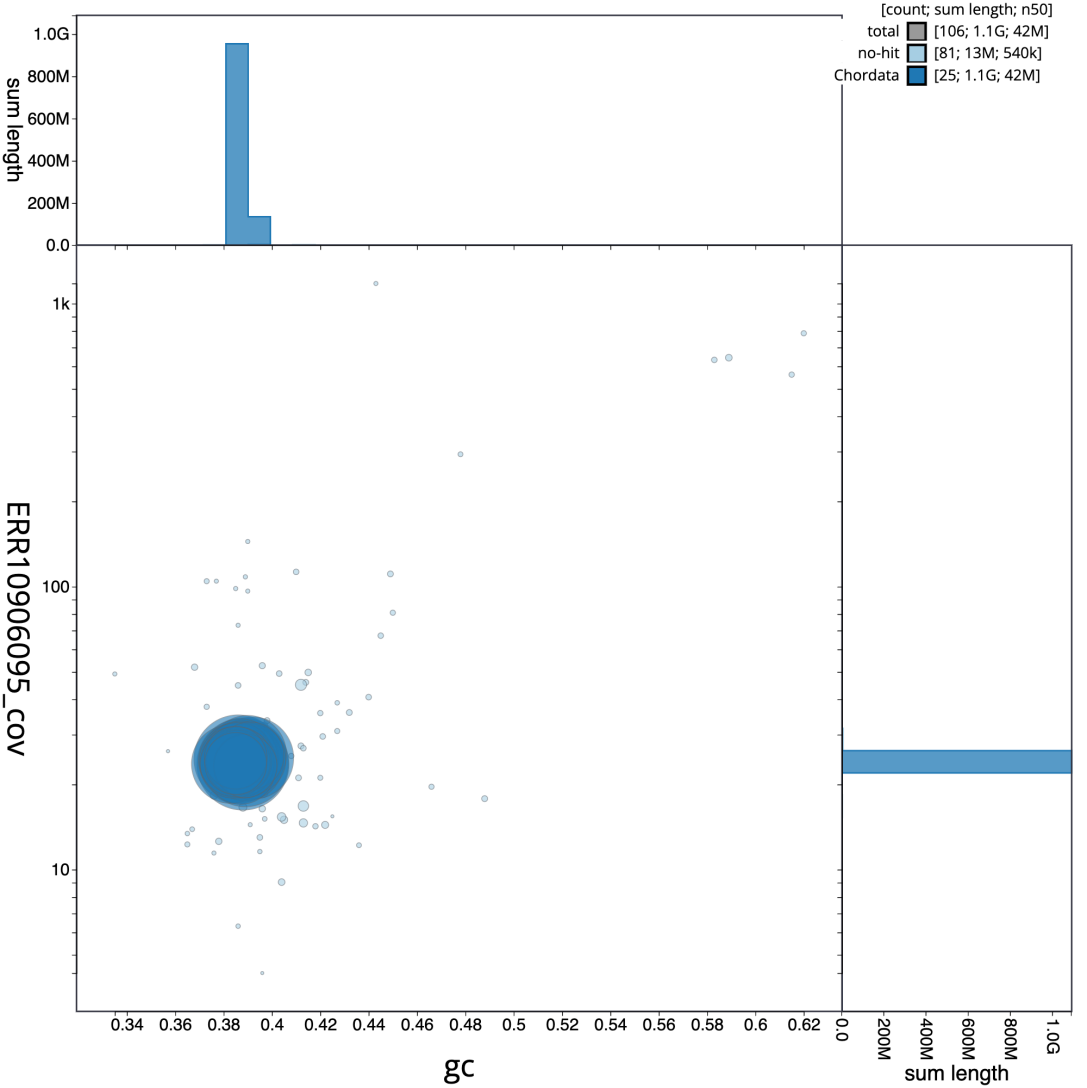
Genome assembly of *Squalius cephalus*, fSquCep2.1: BlobToolKit GC-coverage plot. Scaffolds are coloured by phylum. Circles are sized in proportion to scaffold length. Histograms show the distribution of scaffold length sum along each axis. An interactive version of this figure is available at https://blobtoolkit.genomehubs.org/view/CASGGZ01/dataset/CASGGZ01/blob.

**Figure 4.**
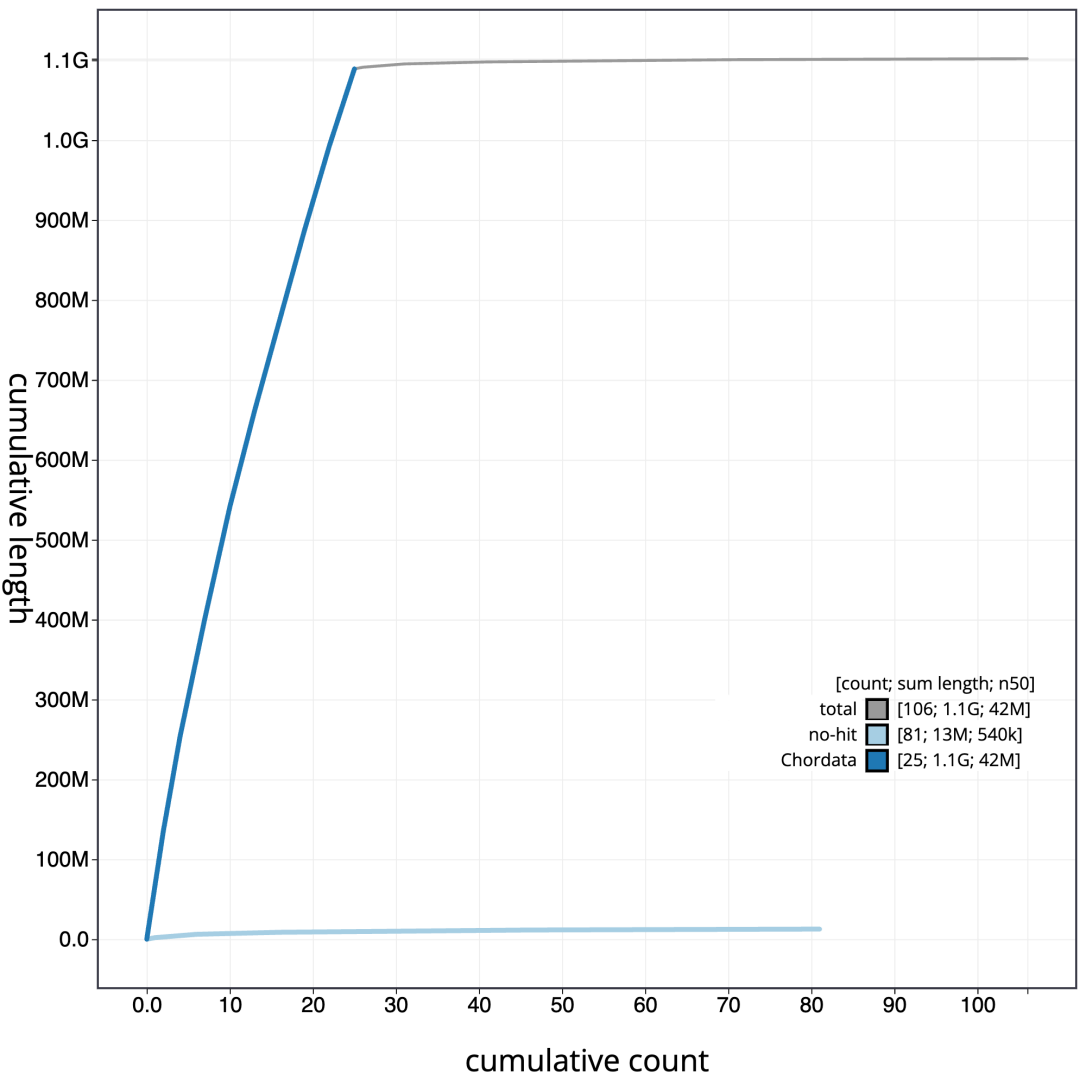
Genome assembly of *Squalius cephalus*, fSquCep2.1: BlobToolKit cumulative sequence plot. The grey line shows cumulative length for all scaffolds. Coloured lines show cumulative lengths of scaffolds assigned to each phylum using the buscogenes taxrule. An interactive version of this figure is available at https://blobtoolkit.genomehubs.org/view/CASGGZ01/dataset/CASGGZ01/cumulative.

**Figure 5.**
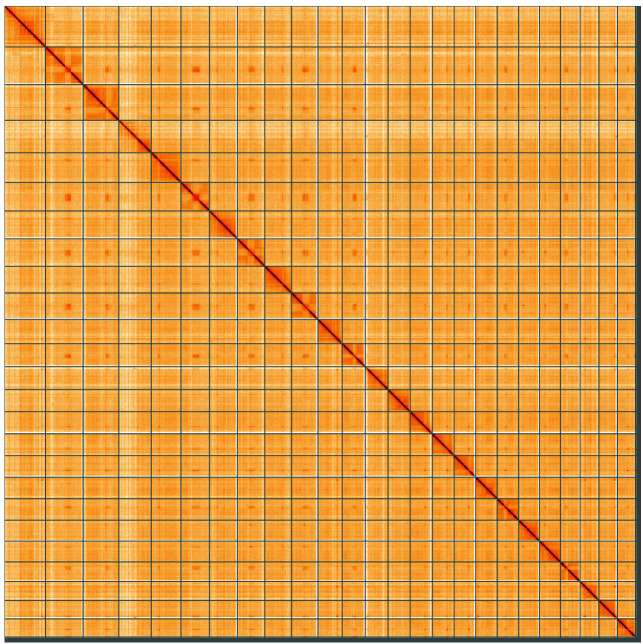
Genome assembly of *Squalius cephalus*, fSquCep2.1: Hi-C contact map of the fSquCep2.1 assembly, visualised using HiGlass. Chromosomes are shown in order of size from left to right and top to bottom. An interactive version of this figure may be viewed at https://genome-note-higlass.tol.sanger.ac.uk/l/?d=UZt60pB1SYOsE72S-xOkNA.

The final assembly has a total length of 1101.9 Mb in 105 sequence scaffolds with a scaffold N50 of 41.8 Mb ( Table 1). The snailplot in Figure 2 provides a summary of the assembly statistics, while the distribution of assembly scaffolds on GC proportion and coverage is shown in Figure 3. The cumulative assembly plot in Figure 4 shows curves for subsets of scaffolds assigned to different phyla. Most (98.84%) of the assembly sequence was assigned to 25 chromosomal-level scaffolds, representing 25 autosomes. Chromosome-scale scaffolds confirmed by the Hi-C data are named in order of size ( Figure 5; Table 2). While not fully phased, the assembly deposited is of one haplotype. Contigs corresponding to the second haplotype have also been deposited. The mitochondrial genome was also assembled and can be found as a contig within the multifasta file of the genome submission.

**Table 1.** Genome data for *Squalius cephalus*, fSquCep2.1.

Project accession data		
Assembly identifier	fSquCep2.1	
Species	*Squalius cephalus*	
Specimen	fSquCep2	
NCBI taxonomy ID	8284	
BioProject	PRJEB59952	
BioSample ID	SAMEA11296547	
Isolate information	fSquCep2	
Assembly metrics *		*Benchmark*
Consensus quality (QV)	58.2	*≥ 50*
*k*-mer completeness	99.99%	*≥ 95%*
BUSCO **	C:97.9%[S:96.3%,D:1.6%], F:0.8%,M:1.3%,n:3,640	*C ≥ 95%*
Percentage of assembly mapped to chromosomes	98.84%	*≥ 95%*
Sex chromosomes	None	*localised homologous pairs*
Organelles	Mitochondrial genome: 16.61 kb	*complete single alleles*
Raw data accessions		
PacificBiosciences SEQUEL II	ERR10906094, ERR10906095	
Hi-C Illumina	ERR10908632	
PolyA RNA-Seq Illumina	ERR11837469	
Genome assembly		
Assembly accession	GCA_949319135.1	
*Accession of alternate haplotype*	GCA_949319155.1	
Span (Mb)	1,101.9	
Number of contigs	694	
Contig N50 length (Mb)	3.0	
Number of scaffolds	105	
Scaffold N50 length (Mb)	41.8	
Longest scaffold (Mb)	70.74	

* Assembly metric benchmarks are adapted from column VGP-2020 of “Table 1: Proposed standards and metrics for defining genome assembly quality” from ( Rhie *et al.*, 2021). ** BUSCO scores based on the actinopterygii_odb10 BUSCO set using version 5.3.2. C = complete [S = single copy, D = duplicated], F = fragmented, M = missing, n = number of orthologues in comparison. A full set of BUSCO scores is available at https://blobtoolkit.genomehubs.org/view/CASGGZ01/dataset/CASGGZ01/busco.

**Table 2.** Chromosomal pseudomolecules in the genome assembly of *Squalius cephalus*, fSquCep2.

INSDC accession	Chromosome	Length (Mb)	GC%
OX439247.1	1	70.74	39.0
OX439248.1	2	64.82	38.5
OX439249.1	3	61.78	39.0
OX439250.1	4	55.87	39.0
OX439251.1	5	51.32	38.5
OX439252.1	6	49.16	39.0
OX439253.1	7	48.07	38.5
OX439254.1	8	47.5	39.0
OX439255.1	9	46.14	38.5
OX439256.1	10	45.67	38.5
OX439257.1	11	41.8	39.0
OX439258.1	12	39.78	38.5
OX439259.1	13	39.4	38.5
OX439260.1	14	38.21	39.0
OX439261.1	15	38.17	38.5
OX439262.1	16	37.59	38.5
OX439263.1	17	37.31	38.5
OX439264.1	18	37.24	39.0
OX439265.1	19	37.11	38.5
OX439266.1	20	36.0	38.5
OX439267.1	21	36.0	38.5
OX439268.1	22	33.75	38.5
OX439269.1	23	32.57	39.0
OX439270.1	24	31.98	38.5
OX439271.1	25	31.21	38.5
OX439272.1	MT	0.02	44.5

The estimated Quality Value (QV) of the final assembly is 58.2 with *k*-mer completeness of 99.99%, and the assembly has a BUSCO v5.3.2 completeness of 97.9% (single = 96.3%, duplicated = 1.6%), using the actinopterygii_odb10 reference set ( *n* = 3,640).

Metadata for specimens, barcode results, spectra estimates, sequencing runs, contaminants and pre-curation assembly statistics are given at https://links.tol.sanger.ac.uk/species/8284.

## Methods

### Sample acquisition and nucleic acid extraction

A *Squalius cephalus* (specimen ID SAN0000713, ToLID fSquCep2) was collected from Calverton Fish Farm, Nottingham, UK (latitude 53.03, longitude –1.05) on 2020-08-21 using a Seine net. The specimen was collected by Richard Pitman (Environment Agency) and identified by Bernd Hänfling (University of Hull), and tissue samples were preserved in liquid nitrogen at –70 °C. The fish was euthanised in benzocaine before being frozen intact. Tissue dissection took place within 30 minutes of euthanasia, and the tissues were immediately shock-frozen in liquid nitrogen and the samples were preserved in liquid nitrogen.

The workflow for high molecular weight (HMW) DNA extraction at the WSI includes a sequence of core procedures: sample preparation; sample homogenisation, DNA extraction, fragmentation, and clean-up. In sample preparation, the fSquCep2 sample was weighed and dissected on dry ice ( Jay *et al.*, 2023). For sample homogenisation, muscle tissue was cryogenically disrupted using the Covaris cryoPREP ^®^ Automated Dry Pulverizer ( Narváez-Gómez *et al.*, 2023). HMW DNA was extracted using the Automated MagAttract v1 protocol ( Oatley *et al.*, 2023). HMW DNA was sheared into an average fragment size of 12–20 kb in a Megaruptor 3 system with speed setting 30 ( Todorovic *et al.*, 2023). Sheared DNA was purified by solid-phase reversible immobilisation ( Strickland *et al.*, 2023): in brief, the method employs a 1.8X ratio of AMPure PB beads to sample to eliminate shorter fragments and concentrate the DNA. The concentration of the sheared and purified DNA was assessed using a Nanodrop spectrophotometer and Qubit Fluorometer and Qubit dsDNA High Sensitivity Assay kit. Fragment size distribution was evaluated by running the sample on the FemtoPulse system.

RNA was extracted from muscle tissue of fSquCep2 in the Tree of Life Laboratory at the WSI using the RNA Extraction: Automated MagMax™ *mir*Vana protocol ( do Amaral *et al.*, 2023). The RNA concentration was assessed using a Nanodrop spectrophotometer and a Qubit Fluorometer using the Qubit RNA Broad-Range Assay kit. Analysis of the integrity of the RNA was done using the Agilent RNA 6000 Pico Kit and Eukaryotic Total RNA assay.

Protocols developed by the Wellcome Sanger Institute (WSI) Tree of Life core laboratory have been deposited on protocols.io ( Denton *et al.*, 2023).

### Sequencing

Pacific Biosciences HiFi circular consensus DNA sequencing libraries were constructed according to the manufacturers’ instructions. Poly(A) RNA-Seq libraries were constructed using the NEB Ultra II RNA Library Prep kit. DNA and RNA sequencing was performed by the Scientific Operations core at the WSI on Pacific Biosciences SEQUEL II (HiFi) and Illumina NovaSeq 6000 (RNA-Seq) instruments. Hi-C data were also generated from muscle tissue of fSquCep2 using the Arima2 kit and sequenced on the Illumina NovaSeq 6000 instrument.

### Genome assembly, curation and evaluation

Assembly was carried out with Hifiasm ( Cheng *et al.*, 2021) and haplotypic duplication was identified and removed with purge_dups ( Guan *et al.*, 2020). The assembly was then scaffolded with Hi-C data ( Rao *et al.*, 2014) using YaHS ( Zhou *et al.*, 2023). The assembly was checked for contamination and corrected as described previously ( Howe *et al.*, 2021). Manual curation was performed using HiGlass ( Kerpedjiev *et al.*, 2018) and PretextView ( Harry, 2022). The mitochondrial genome was assembled using MitoHiFi ( Uliano-Silva *et al.*, 2023), which runs MitoFinder ( Allio *et al.*, 2020) or MITOS ( Bernt *et al.*, 2013) and uses these annotations to select the final mitochondrial contig and to ensure the general quality of the sequence.

A Hi-C map for the final assembly was produced using bwa-mem2 ( Vasimuddin *et al.*, 2019) in the Cooler file format ( Abdennur & Mirny, 2020). To assess the assembly metrics, the *k*-mer completeness and QV consensus quality values were calculated in Merqury ( Rhie *et al.*, 2020). This work was done using Nextflow ( Di Tommaso *et al.*, 2017) DSL2 pipelines “sanger-tol/readmapping” ( Surana *et al.*, 2023a) and “sanger-tol/genomenote” ( Surana *et al.*, 2023b). The genome was analysed within the BlobToolKit environment ( Challis *et al.*, 2020) and BUSCO scores ( Manni *et al.*, 2021; Simão *et al.*, 2015) were calculated.

Table 3 contains a list of relevant software tool versions and sources.

**Table 3.** Software tools: versions and sources.

Software tool	Version	Source
BlobToolKit	4.1.7	https://github.com/blobtoolkit/blobtoolkit
BUSCO	5.3.2	https://gitlab.com/ezlab/busco
Hifiasm	0.16.1-r375	https://github.com/chhylp123/hifiasm
HiGlass	1.11.6	https://github.com/higlass/higlass
Merqury	MerquryFK	https://github.com/thegenemyers/MERQURY.FK
MitoHiFi	2	https://github.com/marcelauliano/MitoHiFi
PretextView	0.2	https://github.com/wtsi-hpag/PretextView
purge_dups	1.2.3	https://github.com/dfguan/purge_dups
sanger-tol/genomenote	v1.0	https://github.com/sanger-tol/genomenote
sanger-tol/readmapping	1.1.0	https://github.com/sanger-tol/readmapping/tree/1.1.0
YaHS	1.2a	https://github.com/c-zhou/yahs

### Wellcome Sanger Institute – Legal and Governance

The materials that have contributed to this genome note have been supplied by a Darwin Tree of Life Partner. The submission of materials by a Darwin Tree of Life Partner is subject to the **‘Darwin Tree of Life Project Sampling Code of Practice’**, which can be found in full on the Darwin Tree of Life website here. By agreeing with and signing up to the Sampling Code of Practice, the Darwin Tree of Life Partner agrees they will meet the legal and ethical requirements and standards set out within this document in respect of all samples acquired for, and supplied to, the Darwin Tree of Life Project.

Further, the Wellcome Sanger Institute employs a process whereby due diligence is carried out proportionate to the nature of the materials themselves, and the circumstances under which they have been/are to be collected and provided for use. The purpose of this is to address and mitigate any potential legal and/or ethical implications of receipt and use of the materials as part of the research project, and to ensure that in doing so we align with best practice wherever possible. The overarching areas of consideration are:

•   Ethical review of provenance and sourcing of the material

•   Legality of collection, transfer and use (national and international)

Each transfer of samples is further undertaken according to a Research Collaboration Agreement or Material Transfer Agreement entered into by the Darwin Tree of Life Partner, Genome Research Limited (operating as the Wellcome Sanger Institute), and in some circumstances other Darwin Tree of Life collaborators.

## Data Availability

European Nucleotide Archive: *Squalius cephalus*. Accession number PRJEB59952; https://identifiers.org/ena.embl/PRJEB59952 ( Wellcome Sanger Institute, 2023). The genome sequence is released openly for reuse. The *Squalius cephalus* genome sequencing initiative is part of the Darwin Tree of Life (DToL) project. All raw sequence data and the assembly have been deposited in INSDC databases. The genome will be annotated using available RNA-Seq data and presented through the Ensembl pipeline at the European Bioinformatics Institute. Raw data and assembly accession identifiers are reported in Table 1.

## Author information

Members of the Darwin Tree of Life Barcoding collective are listed here: https://doi.org/10.5281/zenodo.4893703.

Members of the Wellcome Sanger Institute Tree of Life Management, Samples and Laboratory team are listed here: https://doi.org/10.5281/zenodo.10066175.

Members of Wellcome Sanger Institute Scientific Operations: Sequencing Operations are listed here: https://doi.org/10.5281/zenodo.10043364.

Members of the Wellcome Sanger Institute Tree of Life Core Informatics team are listed here: https://doi.org/10.5281/zenodo.10066637.

Members of the Tree of Life Core Informatics collective are listed here: https://doi.org/10.5281/zenodo.5013541.

Members of the Darwin Tree of Life Consortium are listed here: https://doi.org/10.5281/zenodo.4783558.
