# Supplementary material for: The genome sequence of the European chub, Squalius cephalus (Linnaeus, 1758)
Source: Wellcome Open Res. 2024 Jul 10;9:364. [Version 1] doi: 10.12688/wellcomeopenres.22579.1 (PMC11447678; doi:10.12688/wellcomeopenres.22579.1)
Supplement: Peer review history

# Reviewer report: Reviewer response for version 1

**DOI:** 10.21956/wellcomeopenres.24877.r98175

**Published:** 2024-10-01

**Authors:** Chi-Hing Christina Cheng, Shriram Bhat, Jinmu Kim

**Affiliations:** 1 Department of Evolution, Ecology, and Behavior, University of Illinois Urbana-Champaign, Urbana, Illinois, USA; 2 Evolution Ecology and Behavior, University of Illinois Urbana-Champaign School of Integrative Biology, Urbana, Illinois, USA

I engaged a PhD student and a Postdoctoral researcher in my lab to provide this joint review. I appreciate Wellcome Open Research encourages participation by graduate students and postdocs, providing an invaluable opportunity to gain experience.

***Article assessment***

         The paper reports the sequenced genome of the European chub, *Squalius cephalus, *a widespread freshwater teleost fish, as part of the Darwin Tree of Life Project. HiFi sequencing and Hi-C scaffolding generated a chromosome level assembly with good BUSCO score support, indicating high assembly contiguity and gene completeness. This genome resource will enable further research into the biology of this species and related cyprinids.

***Background***

         A separate chromosome-level genome assembly of *S. cephalus* exists in the public domain; should this be indicated?

***Genome sequence report***

         Replace “from a *Squalius cephalus*…. *” *with “from a *Squalius cephalus* **individual**…..” in the first line. Indicate the sex of the fish if known.

         Also in the first sentence, we suggest removing “(53.03, -1.05)”. The numbers were not labeled with “latitude” and “longitude” and the GPS co-ordinates appear in the first paragraph of the Methods anyway.

         Was there any existing knowledge of the genome size of *S. cephalus*, or cytogenetic data showing the species has a haploid chromosome number of 25?

         The statement that the assembled sequence was assigned to 25 chromosomal-level scaffolds “representing 25 autosomes” needs revision. Unless this chub is asexual, it must have a sex chromosome. Just that the sex chromosome is not morphologically differentiated (the case of the great majorities of teleosts), such that it cannot be distinguished from the 24 autosomes.

         The link metadata link https://links.tol.sanger.ac.uk/species/8284 is inactive. For the review, one would expect it to be active.

***Methods***

         It is a bit odd that RNA was extracted from only a single tissue (muscle) for RNAseq sequencing. One assumes that the purpose of generating the RNAseq data is to annotate the genome. But the manuscript does not include a Genome Annotation Report or a section in Methods on genome annotation. A single tissue transcriptome would be inadequate however for genome annotation.  Please clarify this situation.

         Was the gender, length (TL) and weight of the sequenced chub known? Gender information is particularly important for biologists. The information may be in the metadata file, but it is not accessible to the reviewer.

         There is conflicting information in the sampling of the fish. The Methods state “The fish was euthanised in benzocaine before being frozen intact”, which sounds like the whole fish was frozen post euthanasia. However, the next sentence says, “Tissue dissection took place within 30 minutes of euthanasia, and the tissues were immediately shock-frozen in liquid nitrogen….”. Was the actual sequence of events as follows - fish euthanized, tissued dissected with 30 min., tissues and the remaining carcass flash frozen in LN2? Please clarify/revise.

         For reproducibility, it would be useful to indicate whether the HiFiasm run used default parameters or used additional parameters such as Hi-C integration.

         The bwa-mem2 tool is an aligner, and does not generate Hi-C maps, thus the sentence “A Hi-C map for the final assembly was produced using bwa-mem2” will confuse readers.

**Is the rationale for creating the dataset(s) clearly described?**

         Yes. The mention the goal of the project was to add the newly sequenced genome to the Darwin Tree of Life Project.

**Are the protocols appropriate and is the work technically sound?**

         Yes.

**Are sufficient details of methods and materials provided to allow replication by others?**

         Yes, except for the specific comments on sampling, the “orphan” RNAseq effort, and a couple of bioinformatics items. There are also instances where more detailed descriptions of the computational analyses would be particularly helpful for biologists try to assemble genome data, but we understand the page limit of the DATA NOTE.

**Are the datasets clearly presented in a useable and accessible format?**

      Yes, the dedicated section for data availability ensures easy access of the published genome.

Are sufficient details of methods and materials provided to allow replication by others?

Partly

Is the rationale for creating the dataset(s) clearly described?

Yes

Are the datasets clearly presented in a useable and accessible format?

Yes

Are the protocols appropriate and is the work technically sound?

Yes

Reviewer Expertise:

Evolutionary genomics and transcriptomics of cold adaptation in polar fishes and cold hardy insects, HiFi and RNA sequencing; Genomics; Bioinformatics; Evolution

We confirm that we have read this submission and believe that we have an appropriate level of expertise to confirm that it is of an acceptable scientific standard.

# Reviewer report: Reviewer response for version 1

**DOI:** 10.21956/wellcomeopenres.24877.r94203

**Published:** 2024-09-02

**Authors:** Hiranya Sudasinghe

**Affiliations:** 1 University of Bern, Bern, Switzerland

This Data Note presents a high-quality genome assembly of *Squalius cephalus*, a widely distributed cyprinid species in Europe belonging to the family Leuciscidae. The bioinformatic pipeline utilized in this assembly follows the standard methodologies adopted by the Darwin Tree of Life Project. The genome assembly exceeds the recommended benchmarks for quality and completeness.

 While the primary aim of the Darwin Tree of Life Project is to sequence all named eukaryotic species in the Atlantic Archipelago of Britain and Ireland, the authors could strengthen the Introduction by providing additional biological and economic insights into the significance of sequencing the genome of *Squalius cephalus*.

 It is also noteworthy that there are already a few genome assemblies of *Squalius* species available in NCBI. This newly curated assembly could further facilitate comparative genomic studies within this group, such as those aimed at understanding ploidy evolution in *Squalius* and related taxa.

 Minor comments

 1. In the Abstract, *Squalius cephalus* is incorrectly classified under the family Cyprinidae. This should be corrected to Leuciscidae.

 2. If possible, it would be beneficial to include information about the sex of the sequenced specimen in the manuscript.

 3. During the assembly step with hifiasm, it would be helpful to clarify whether Hi-C data was incorporated into the assembly process itself or if it was solely used in the scaffolding step.

 4. In the mitochondrial genome extraction section, it is stated that "The mitochondrial genome was assembled using MitoHiFi (Uliano-Silva et al., 2023), which runs MitoFinder (Allio et al., 2020) or MITOS (Bernt et al., 2013) and uses these annotations to select the final mitochondrial contig and to ensure the general quality of the sequence." Please clarify whether both MitoFinder and MITOS were run for the assembly. By default, MitoHiFi uses only MitoFinder for annotation

 5. The Methods section mentions the preparation of an RNA library; however, there is no subsequent information on the use of RNA data for genome annotation.

Are sufficient details of methods and materials provided to allow replication by others?

Partly

Is the rationale for creating the dataset(s) clearly described?

Yes

Are the datasets clearly presented in a useable and accessible format?

Yes

Are the protocols appropriate and is the work technically sound?

Yes

Reviewer Expertise:

Phylogenomics; Ichthyology; Systematics; Comparative genomics

I confirm that I have read this submission and believe that I have an appropriate level of expertise to confirm that it is of an acceptable scientific standard.
